# Radiation Induced Enhancement of Hydrogen Influence on Luminescent Properties of nc-Si/SiO_2_ Structures

**DOI:** 10.1186/s11671-016-1744-7

**Published:** 2016-12-07

**Authors:** Igor Lisovskyy, Mariia Voitovych, Volodymyr Litovchenko, Vasyl Voitovych, Iurii Nasieka, Viktor Bratus

**Affiliations:** 1 V.Ye. Laskarev Institute of Semiconductor Physics, National Academy of Sciences of Ukraine, prosp. Nauki 41, Kyiv, 03028 Ukraine; 2 Institute of Physics, National Academy of Sciences of Ukraine, prosp. Nauki 46, Kyiv, 03028 Ukraine

**Keywords:** Si nanocrystals, SiO_2_ films, Photoluminescence, Infrared spectroscopy, Electron-spin resonance, *γ*-irradiation, Radiation defects

## Abstract

Using photo-luminescence, infrared spectroscopy, and electron spin resonance technique, the silicon dioxide films with embedded silicon nanocrystals (nc-Si/SiO_2_ structures) have been investigated after *γ*-irradiation with the dose 2 × 10^7^ rad and subsequent annealing at 450 °C in hydrogen ambient. For the first time, it was shown that such a radiation-thermal treatment results in significant increase of the luminescence intensity, in a red shift of the photoluminescence spectra, and in disappearance of the electron-spin resonance signal related to silicon broken bonds. This effect has been explained by passivation of silicon broken bonds at the nc-Si–SiO_2_ interface with hydrogen and by generation of new luminescence centers, these centers being created at elevated temperatures due to transformation of radiation-induced defects.

## Background

Optimization of the emission characteristics (intensity enhancement and widening the emission spectral range) of the nc-Si/SiO_2_ structures remains a topical task yet. According to this aim, during the last decades, many researchers have directed their investigations on the study of the influence of the formation techniques as well as post-growth treatments on the structural and luminescent properties of these film systems. Taking into account the results of numerous investigations, one can summarize that just changes in the impurity-defect state of the nc-Si/SiO_2_ structure are able to substantially influence on its luminescent characteristics. Herewith, the most sensitive to effect of doping atoms and structural defects (similar to microelectronic planar system Si/SiO_2_) is the nc-Si/oxide interface. In general, this effect is caused by a wide range of physical processes–from passivation of the broken Si chemical bonds (quenching of the non-radiative recombination channels) to formation of the complex defects that are able to mediate the radiative recombination processes (formation of radiative recombination channels). Also, this effect depends on several factors: type of impurities, sizes of impurity atoms, solubility, and ability to form stable chemical bonds with silicon or oxygen. These impurities may be introduced at the stage of nc-Si/SiO_2_ structure formation [1, 2] as well as due to its low-temperature treatment in the ambient atmosphere of chemically active gas (hydrogen, nitrogen, or oxygen) [3–9]. Influence of these thermal treatments on radiative recombination in nc-Si depends on chemical composition of the annealing environment, temperature, and duration. Annealing in the ambient atmosphere that includes hydrogen is the most efficient [5–8].

The defect-impurity state of the nc-Si surface and parameters of light emission, respectively, may be changed by ionizing radiation treatment. For instance, irradiation of porous silicon samples with *γ*-quanta (with the dose 4.3 × 10^6^–3 × 10^8^ rad) resulted in the remarkable (up to five times) increase of the red luminescent band near 710 nm (~1.75 eV) and in its blue shift [10]. Irradiation with a larger dose (10^10^ rad) led to *I*_PL_ decrease (up to 40 times) [11]. It is important to note that this *I*_PL_ enhancement took place due to radiation treatment in air atmosphere only [12] and was explained by porous silicon surface oxidation during irradiation that created around the sample corrosive ionized ambient containing ozone, atomic hydrogen, and oxygen.

Certain enhancement (~1.5 times) of the luminescent intensity was observed earlier [13] after low-dose (~10^4^ rad) ionizing irradiation (with *γ*-rays) of SiO_2_ films containing Si nanocrystals. Contrary to the case of porous silicon, effect in the nc-Si/SiO_2_ structures was observed after radiation treatment even in inert gas or vacuum; hence, it had a different nature. Recently, the authors of [14] reported that proton irradiation of nc-Si in SiO_2_ multilayers leads to significant increase in the intensity of the luminescence centered near 750 nm, in blue shift of the photoluminescence spectra, and in appearance and increase of the luminescence band near 500 nm. Infrared spectroscopy results demonstrated a decrease in the absorption peak corresponding to SiO_2_.

On the other hand, irradiation with rather high doses (~10^5^–10^6^ rad) of the semiconductor-dielectric systems is known to substantially facilitate elimination of surface recombination defects under following thermal treatments resulting in markedly improvement of electrical (Si/SiO_2_ systems) and luminescent (GaAs/oxide, InSb/oxide systems) characteristics [15]. Therefore, one may expect that irradiation and subsequent thermal treatment of nc-Si/SiO_2_ structures are also able to enhance their light-emitting efficiency. To check this hypothesis, the investigation has been done, in which the influence of high-dose γ–irradiation followed by low-temperatures annealing on luminescent properties of nc-Si/SiO_2_ structures was studied.

## Methods

SiO_x_ layers were obtained by thermal evaporation of SiO (Cerac Inc., purity of 99.9%) in vacuum at the residual pressure of 2 × 10^−3^ Pa. Both-side polished *p*-Si wafers with the resistivity of 10 and 50 Ohm cm^−1^ were used as substrates. The substrate temperature during deposition was 150 °C. The film thickness (*d*) was estimated in situ by the quartz-crystal-oscillator monitor system (KIT-1) and was 450 nm (on the substrate with *ρ* = 10 Ohm cm^−1^ for photo-luminescence and infrared measurements) or 1000 nm (on the substrate with *ρ* = 50 Ohm cm^−1^ for electron-spin resonance measurements). After deposition, it was measured with the MII-4 microinterferometer and profilometer Dectak 3030. In the result of subsequent high-temperature (*T* = 1100 °C) thermal treatment in Ar atmosphere for 15–30 min, the nc-Si/SiO_2_ structures were formed. The previous investigations of such samples using transmitting electron microscope of high resolution [16] have shown that the average size of the formed nanocrystallites is about 3 nm, and they are sufficiently evenly distributed through the film.

These structures have been irradiated by *γ*-quanta (^60^Co) with the intensity 36.77 rad s^−1^ and the energies 1.17 and 1.33 MeV. The dose of exposure was 2 × 10^7^ rad. The temperature of the samples during irradiation did not exceed 30 °C. Initial and irradiated samples were thermally treated at the temperature of 450 °C in H_2_ atmosphere for 2 h.

Infrared (IR) transmission spectra were measured using Fourier transformed IR spectrometer Spectrum BXII PerkinElmer. A silicon substrate (without oxide film) served as the reference sample. The absorption band related to Si–O bonds (maximum position within the range of 1000–1100 cm^−1^ depending on the oxygen content in the oxide film) was under investigation.

To investigate luminescent properties of the studied samples, the Raman and photoluminescent spectrometer HORIBA Jobin-Yvon T64000 was used. As an excitation tool for photoluminescence, the 488 nm (2.54 eV) line of Ar-Kr laser was applied. Photoluminescence (PL) measurements were carried out at room temperature and at various excitation powers. Taking into account that the measured red luminescence band (maximum position in the vicinity of 800 nm) for initial or annealed in hydrogen ambient samples was not symmetrical; therefore, its deconvolution into elementary profiles has been carried out. To obtain more reliable results, the well-known approach [17] was used. According to this method, one should measure the PL spectra several times under different conditions selected in such a manner that contribution of each component was different in every case but the kind of profile and their main parameters (maximum position and full width at half maximum) were kept constant. The number of measured spectra should exceed that of individual components. Preliminary measurements showed that, in the case of initial or annealed nc-Si/SiO_2_ structures, the shape of PL spectra sufficiently depended on the excitation light intensity (*I*_ex_); therefore, we measured a set of PL spectra under different values of *I*_ex_ (within the range 10^17^–10^19^ quanta cm^−2^ s^−1^). Then the spectral curves for each kind of treatment were deconvoluted into Gaussian profiles with the stable parameters. The deconvolution accuracy was characterized by the root-mean-square deviation of the summed Gaussian profiles from the experimental curve and in these experiments did not exceed the value 10^−2^.

Electron-spin resonance (ESR) spectra were measured at the temperatures *T* = 300 К and *T* = 77 К using the samples of nc-Si/SiO_2_ structures with the substrates of *p*-Si (*ρ* = 50 Ohm cm^−1^) and with the oxide matrix thickness ~1 μm. The measurements were provided in X-band (the frequency of microwaves *ν*~9.4 kHc), magnetic field being modulated at the frequency of 100 kHz. The microwave power and amplitude of magnetic field modulation did not exceed the values of 1 mW and 0.06 mT, respectively. To avoid the effects of saturation and overmodulation, when registering the rather narrow and weak ESR lines, the averaging of 16 to 49 measured spectra was carried out. The number of paramagnetic defects and the value of *g*-factor were determined using the reference MgO:Mn^2+^ sample with the known number of spins, this sample being located within the microwave resonator simultaneously with the sample under investigation. The amount of defects was determined comparing the double integrals of the first derivatives for absorption signals inherent to the sample under study and the reference one. The absolute error in determination of defects amount was ±40%; the relative error, when comparing different samples did not exceed 15%.

## Results and Discussion

Thermal treatments of the initial nc-Si/SiO_2_ samples in inert (Ar) atmosphere at 450 ^o^C did not influence on the PL intensity (*I*_PL_). After irradiation that has decreased the original value of *I*_PL_ about two times, this annealing only restored *I*_PL_ magnitude, which was expected and evidently related to elimination of radiation damage.

**Fig. 1:**
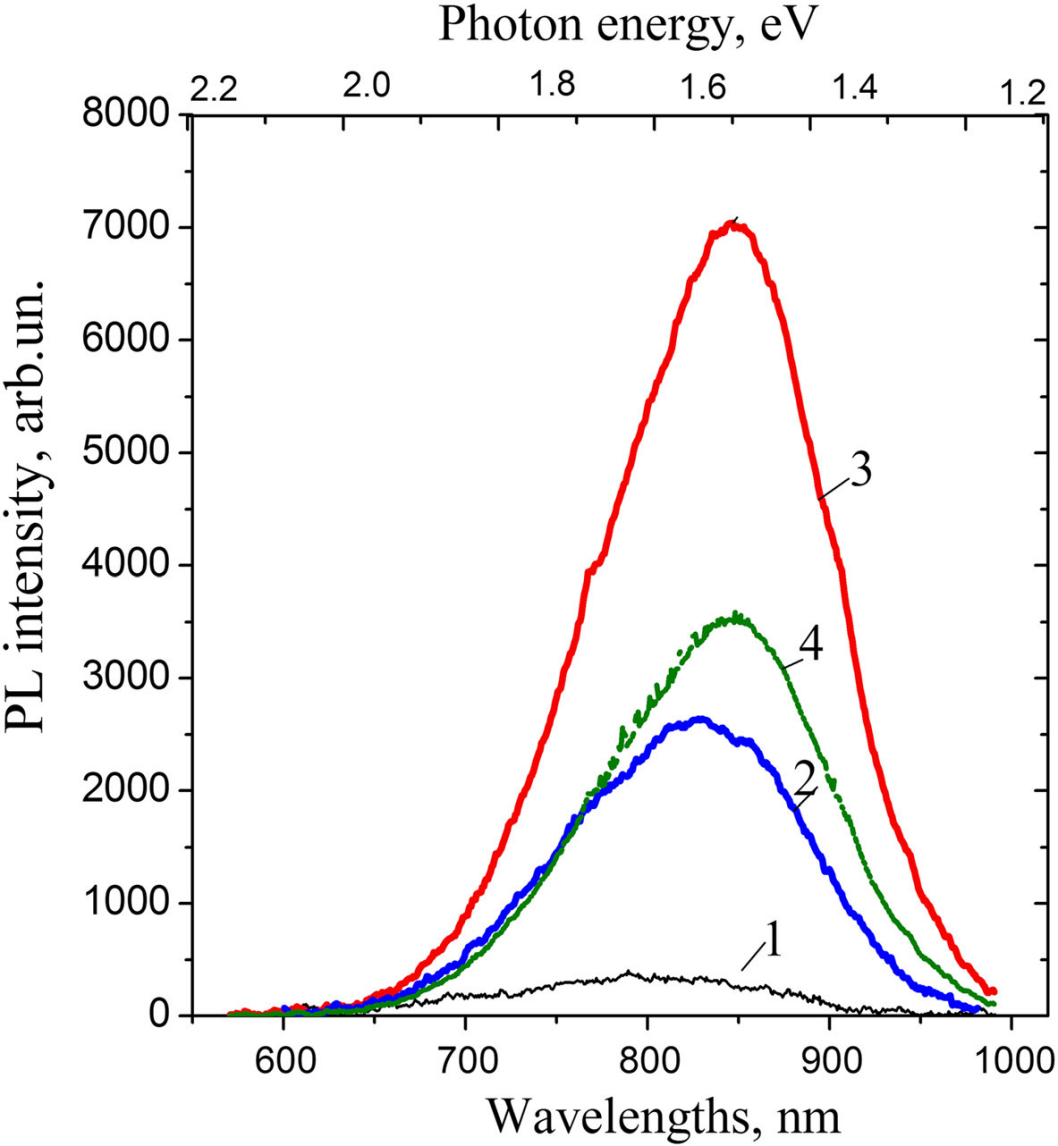
PL spectra of the investigated nc-Si/SiO_2_ structures. **1** Initial sample. **2** Structure thermally treated in hydrogen. **3** Sample annealed in hydrogen within 1 day after irradiation. **4** Sample annealed within 3 years after irradiation

In Fig. 1, the PL spectra of nc-Si/SiO_2_ structures annealed in hydrogen ambient are presented. It is seen that the thermal treatment of initial samples (Fig. 1, curve 2) leads to the ~5-fold increase in the PL intensity, which is in good agreement with the known results [5–8]. In the case of preliminary irradiated samples, the influence of the hydrogen annealing on the *I*_PL_ value was much stronger depending on the time lapse between irradiation and annealing processes. If the latter was rather short (days or weeks) the PL intensities for irradiated and unexposed samples differ up to 2.5 times (Fig. 1, curve 3). In the case when irradiated samples have been annealed within a few years this difference decreases to~1.5 times (Fig. 1, curve 4). From the results presented in Fig. 1, one more observation should be made–the maximum position of PL band for the irradiated and annealed sample is red shifted (~30–50 nm) as compared with that of initial film. Thus, radiation and subsequent low-temperature treatments in hydrogen ambient of nc-Si/SiO_2_ structures result in a new effect–essential enhancement of the PL intensity and the red shift of the PL band.

**Fig. 2:**
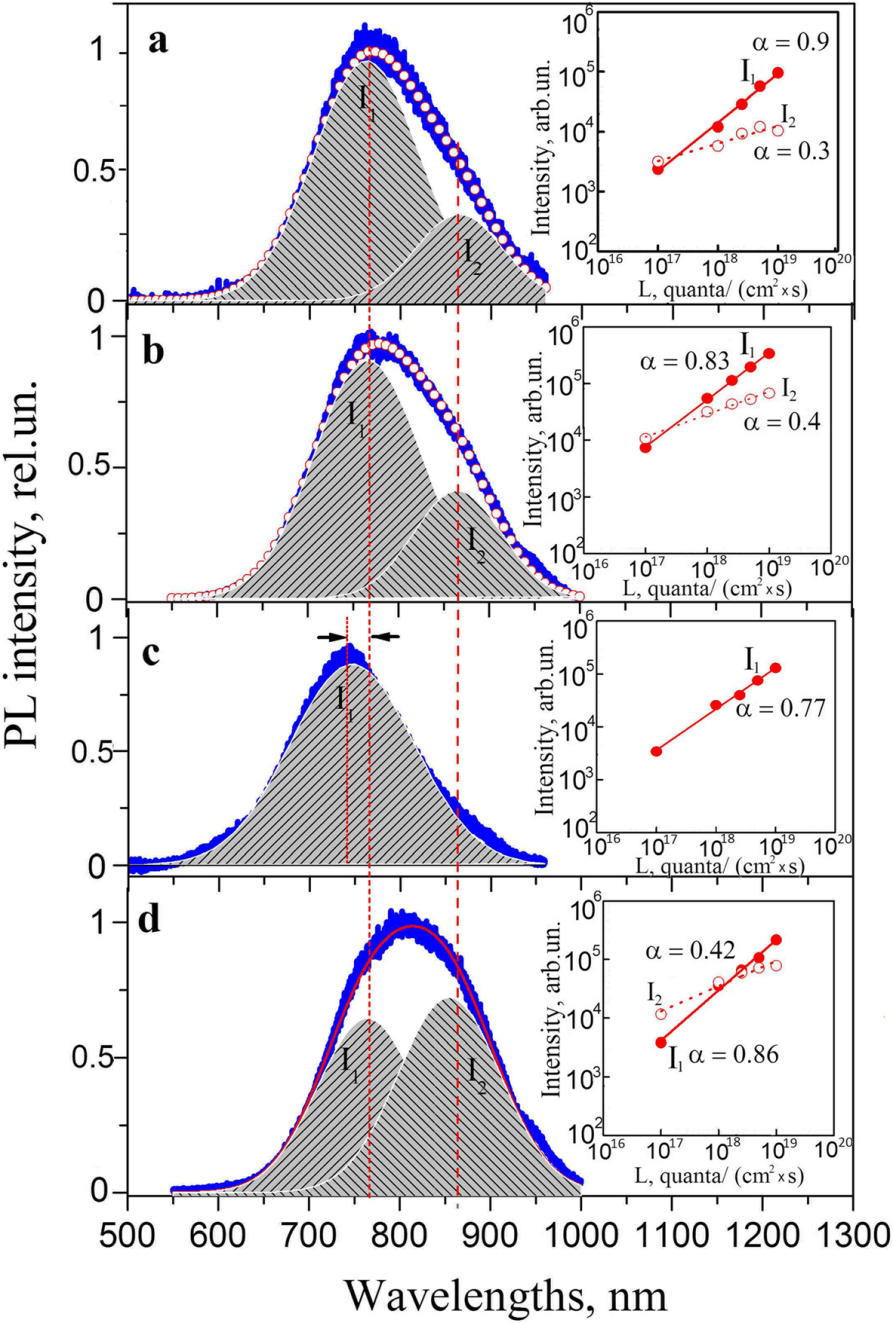
Deconvolution of PL spectra into Gaussian profiles for initial (**a**), thermally treated in hydrogen (**b**), *γ*-irradiated with the dose 2 × 10^7^ rad (**c**), and *γ*-irradiated and thermally treated in hydrogen nc-Si/SiO_2_ structures (**d**). PL spectra were measured at the maximum excitation intensity (10^19^ quanta cm^−2^ s^−1^). In the inserts, the excitation power dependences of the area of corresponding Gaussians are displayed

To obtain additional information on the mechanisms of the observed phenomena, the analysis of PL band and its behavior due to carried treatments has been done. It turns out that the studied red PL band consists of two stable Gaussian profiles *I*_1_ and *I*_2_ with peak positions at *λ*_1_ = 755 ± 5 nm and *λ*_2_ = 863 ± 3 nm and with full widths at half maximum *w*_1_ = 120 ± 5 nm and *w*_2_ = 100 ± 5 nm, respectively (Fig. 2). Contribution of each component to the general PL band depends on the type of sample treatment and power of excitation light. For example, for the non-irradiated (initial and hydrogen annealed) structures the elementary component *I*_1_ was always dominant. Its contribution to the general PL band (*S*_1_/*S*, where *S*_1_ and *S* are the areas of the *I*_1_ profile and the general red PL band, respectively) slightly (~10%) decreases, when the excitation power decreases by two orders of magnitude. At the same time, contribution of the weak *I*_2_ component (*S*_2_/*S*, where *S*_2_ is the area of the *I*_2_ profile) slightly increases. Irradiation of the samples leads to disappearance of the *I*_2_ component. The following thermal treatment induces reconstruction of the abovementioned PL band, but now behavior of these contributions becomes fundamentally different in comparison with that in non-irradiated samples. At a decrease of the excitation power by two orders of magnitude, contribution of the *I*_1_ profile falls by more than two times. Thus, contribution of the *I*_2_ profile becomes dominant even at the excitation level equal to ~0.1 of the maximum one (10^19^ quanta cm^−2^ s^−1^), and when decreasing the excitation power down to 0.001 of the maximum value only the *I*_2_ profile remains.

**Fig. 3:**
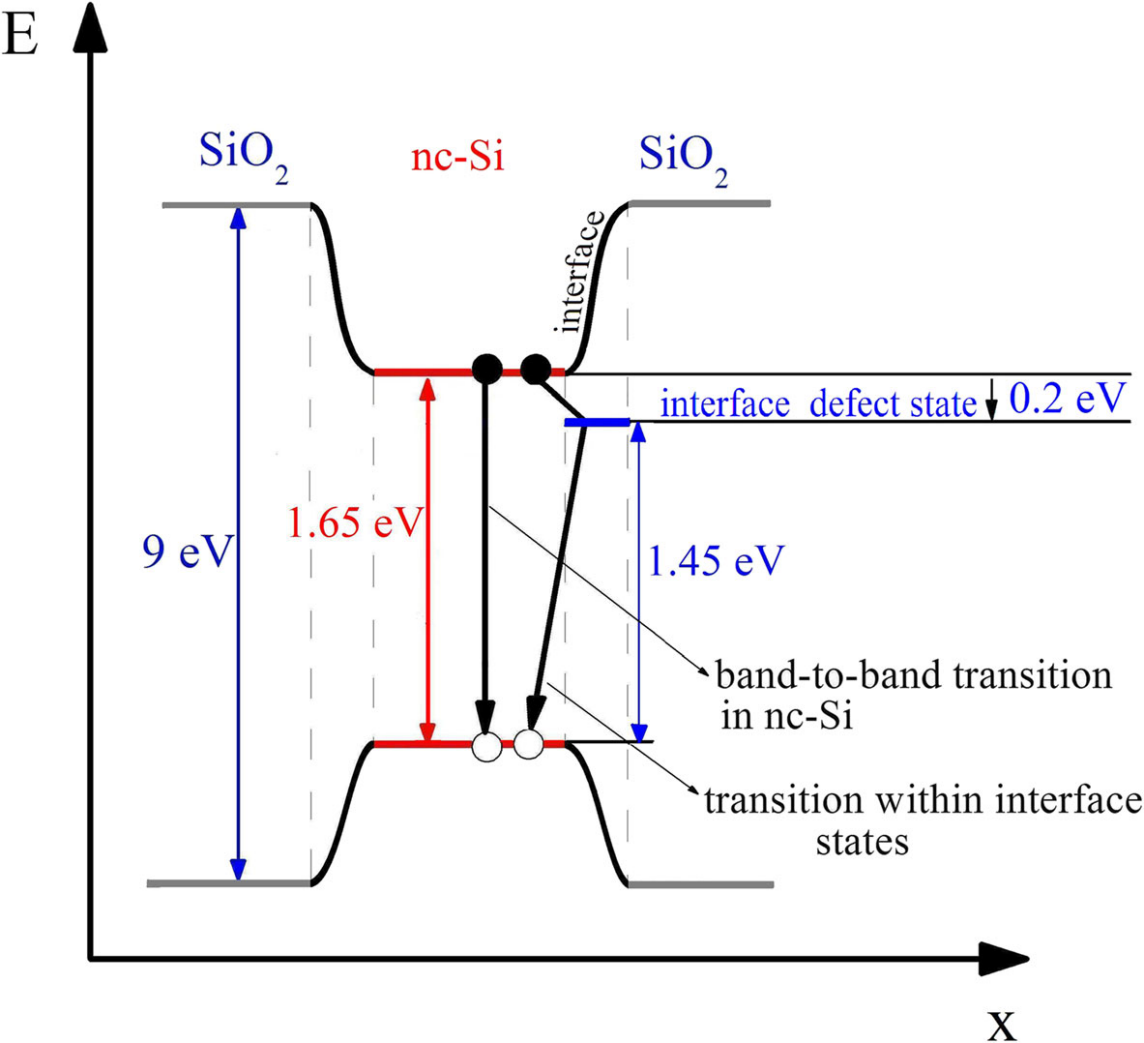
Scheme of radiative recombination in the irradiated and annealed nc-Si/SiO_2_ structure

In Fig. 2, the dependences of intensity of both PL contributions on the excitation level are adduced. Usually, the dependence of the PL intensity (*I*) on the excitation power (*P*) is approximated by a power law function *I = P* ^*α*^, where *α* (slope coefficient) is an exponent that is often used to identify the origin of emission from semiconductors: *α~1* for exciton-like transition and *α* < <1 for free-to-bound and donor–acceptor pair transitions [18]. In fact, we ascertained sublinear dependences with the slopes of 0.9 and 0.3 for *I*_1_ and *I*_2_ components, respectively. This result allows us to conclude that in the studied samples two luminescence mechanisms with different nature occur, and contribution of these mechanisms depends on the sample treatment type. Moreover, the value *α*_1_ (close to 1) allows to conclude that the *I*_1_ component is caused by the band-to-band recombination of the electron-hole pairs in the Si nanocrystals. This mechanism, attributed to quantum confinement effect, is considered as the major one, when explaining the emission in the Si nanocrystallites both in porous Si and incorporated into SiO_2_ film [19]. The obtained value of *λ*_1_ ≈ 755 nm in our case allows to estimate the energy band gap of nc-Si, which is Δ*E ≈* 1.65 eV. Taking into account that the average size of Si nanocrystallites embedded into the studied oxide films is equal to~3 nm, the above ascertained value ΔE well agrees with calculation results as well as with experimental data (see, for example [20]). The substantially lower value *α*_2_ *≈* 0.3 can be attributed to the radiative transitions with participation of the interface defect states in the nc-Si/SiO_2_ transition region [18, 21]. Also, the value of *λ*_2_ ≈ 860 nm allows to estimate the energy position of these interface defect states–it is close to 0.2 eV relatively to the corresponding edge of the nc-Si band gap. In Fig. 3, we schematically showed recombination processes responsible for PL emission in the studied samples.

**Fig. 4:**
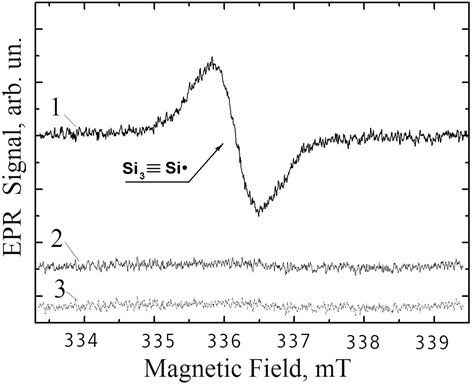
ESR spectra of defects for initial (**1**), annealed in hydrogen (**2**), *γ*-irradiated and thermally treated in hydrogen (**3**) nc-Si/SiO_2_ structures

Thus, light emission in the non-irradiated (both initial and hydrogen annealed) nc-Si/SiO_2_ structures was realized mainly as a result of radiative recombination of electron-hole pairs in the Si nanocrystallites. The minor part of PL can be explained by radiative recombination through the defect centers at the nc-Si–SiO_2_ interface. The enhancement of PL of the nc-Si/SiO_2_ structures after low-temperature annealing in the hydrogen ambient is usually explained by hydrogen atom passivation of the silicon broken bonds (nonradiative recombination centers) that exist in a high concentration at the surface of Si nanocrystallites [22]. In Fig. 4, the ESR spectra for our samples have been shown. It is seen that the band corresponding to silicon broken bonds (including P_b_-centers) with *g* = 2.0058 disappears after thermal treatment in hydrogen ambient both of initial and irradiated nc-Si/SiO_2_ samples.

This fact enabled us to infer that the process of silicon broken bonds passivation by hydrogen atoms was completed practically with the same efficiency in both cases. In other words, passivation of silicon broken bonds due to hydrogen annealing of irradiated structures takes place, but can not be the reason of the enhanced PL intensity.

**Fig. 5:**
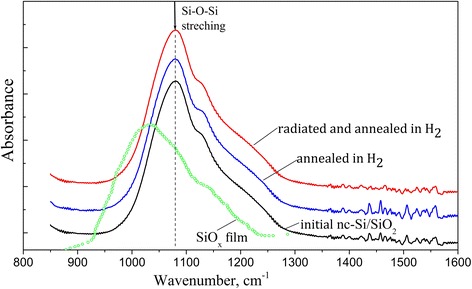
IR spectra of SiO_x_ film and nc-Si/SiO_2_ samples registered at room temperature

Hence, to clarify this effect one should take into account other additional mechanisms, effective factor of which should be interaction of ionizing radiation with the components of the nc-Si/SiO_2_ nanocomposite. It is well known, for example, that the intense ionizing irradiation of the silicon oxide films in vacuum can lead to formation of Si nanocrystallites due to the effect of radiation induced reduction of SiO_2_ [23, 24]. In principle, formation of additional nc-Si should lead to enhancement of the PL intensity. However, in our case the studied samples were irradiated in air and the irradiation power was not high. Furthermore, the IR absorption band related to stretching vibrations of oxygen atoms in Si–O–Si units (maximum position near ~1090 cm^−1^) with the shape inherent to SiO_2_ phase [25] practically did not vary after annealing both the initial and irradiated nc-Si/SiO_2_ samples in inert or hydrogen environment (see Fig. 5). This fact should mean that thermal treatments did not affect the concentration and structural arrangement of the bridging oxygen atoms in the oxide matrix. In other words, this processing did not additionally increase the elemental silicon content in the sample, similarly, for example, to high temperature treatment of the initial SiO_x_ films (see Fig. 5). Hence, the growth of PL intensity observed after radiation-thermal treatments of nc-Si/SiO_2_ structures can not be explained by the growth of the number of silicon nanoinclusions.

The analysis of the dependences of PL intensities on the excitation level has shown that, in the case of annealing the irradiated structures, the role of radiative recombination through the defect states is significantly increased. It is logical to conclude that radiation-thermal treatments lead to the increase in the concentration of defect centers, the energetic levels of which are rather close to the edges of the optical gap in the nanocrystallites. Recombination of charge carries with participation of the mentioned defects provides excess (in comparison with non-irradiated samples) light emission. The energy of these quanta is slightly (~0.2 eV) lower than the width of the optical gap, which in fact causes the observed PL band red shift.

In literature, the authors have reported several candidates on the role of surface defects which can create similar radiative recombination states: ESR centers *P*′_ce_ and *P*′_h_, concentration of which correlates with the intensity of the red PL band [26], silanol groups Si = O [27], complexes SiH_*x*_ (*x* = 1, 2) [28]. In our case, the latter can be formed under the thermal treatment in hydrogen ambient in excess (in comparison with non-irradiated structures) concentrations, because the most probable radiation-induced defects that form in the region of Si–SiO_2_ interface, are the broken silicon bonds Si^3+^ [29]. However, the above mentioned surface defects were invoked to explain the relatively low emission energy of small Si nanocrystallites (with the sizes less than ~2.8 nm [28]), when according to the quantum-confinement effect the width of the optical gap increases, so that their energy states fall near its edges. In our case (rather large nanocrystals), it does not take place.

The observed changes in the emission of the nc-Si/SiO_2_ structures as a result of radiation-thermal treatment (increase in the intensity and the red shift of PL band, as well as their dependence on the time interval between the exposure and annealing), we explain in the following way. The ionizing radiation leads to the substantial damage at the Si-nanocrystals/oxide interface region: (i) the broken Si bonds (as efficient non-radiative recombination centers) are generated, (ii) the radiative recombination centers disappear, and (iii) some metastable defects (that can be partially eliminated even at room temperature) are created. The latter are not light emitting and electrically active, may be due to their energy levels that are not located within nc-Si optical gap; hence, they don’t affect the red PL band. However, the processes (i) and (ii) cause its remarkable quenching. The subsequent low-temperature thermal treatment in hydrogen ambient leads to passivation of the silicon broken bonds (enhancing the intensity of the *I*_1_ contribution) and (that is the key) gives rise to transformation of oxide structural arrangement in the vicinity of metastable defects, thus varying their energetics in consequence of, e.g., the change in the mechanical stresses. As a result, the energy level of the metastable defects is shifted and falls inside the nc-Si optical gap, permitting them to take part in radiative recombination. This involves the considerable growth of the intensity of *I*_2_ contribution and corresponding red shift of PL band.

What is the nature of the metastable defects and what is the role of hydrogen in structural transformations remains unknown yet. Recently, we have obtained preliminary results which demonstrate a strong enhancement of the red PL band of the exposed nc-Si/SiO_2_ structures after annealing even in the inert ambient; however, a special temperature regime of the thermal treatment is necessary. An additional research enables to make a specific conclusion on the mechanism of the radiation-thermal treatment.

## Conclusions

Thus, summarizing the presented experimental results we can conclude that thermal annealing in the hydrogen ambient of *γ*–irradiated nc-Si/SiO_2_ structures leads to the substantial (more than two times in comparison with the annealed initial samples) increase in the photoluminescence intensity, causes the red shift (up to ~50 nm) of the PL band, quenches the ESR signal, attributed to broken Si bonds (including *P*_b_-centers). These effects were explained by passivation of silicon broken bonds on the surface of Si nanocrystals with hydrogen and by generation of new light-emitting centers. These centers are the most probably formed as a result of thermally stimulated transformation of radiation-induced defects at the nc-Si–SiO_2_ interface. Their nature and mechanism of creation require further investigations and discussion.

## Abbreviations

ESR: Electron spin resonance
FTIR: Fourier transform infrared spectroscopy
IR: Infrared
nc-Si/SiO_2_: The silicon dioxide films with embedded silicon nanocrystals
PL: Photoluminescence
TEM: Transmission electron microscopy.

## References

[1] Wilkinson AR, Elliman RG (2004). The effect of annealing environment on the luminescence of silicon nanocrystals in silica. J Appl Phys.

[2] Sopinskyy MV, Vlasenko NA, Lisovskyy IP (2015). Formation of nanocomposites by oxidizing annealing of SiOx and SiOx<Er, F>films: ellipsometry and FTIR analysis. Nanoscale Res Lett.

[3] Bineva I, Nesheva D, Aneva Z (2003). Effects of annealing atmosphere and Raman scattering from Si nanocrystals in a SiO_2_ matrix. J Mater Sci Mater Electron.

[4] Wu X, Beck A, Bittner AM (2003). The effect of annealing conditions on the red photoluminescence of nanocrystalline Si/SiO_2_ films. Thin Solid Films.

[5] Cheylan S, Elliman RG (2001). Photoluminescence from Si nanocrystals in silica: the effect of hydrogen. Nucl Instr Meth B.

[6] Wilkinson AR, Elliman RG (2003). Passivation of Si nanocrystals in SiO_2_: atomic versus molecular hydrogen. Appl Phys Lett.

[7] Lopez M, Garrido B, Garcia C (2002). Elucidation of the surface passivation role on the photoluminescence emission yield of silicon nanocrystals embedded in SiO_2_. Appl Phys Lett.

[8] Pellegrino P, Garrido B, Garcia C (2003). Enhancement of the emission yield of silicon nanocrystals in silica due to surface passivation. Phys E.

[9] Khatsevich I, Melnik V, Popov V (2008). Effect of low temperature treatments on photoluminescence enhancement of ion beam synthesized Si nanocrystals in SiO_2_ matrix. Semicond Phys Quantum Electron Optoelectron.

[10] Fu JS, Mao JC, Wu E (1993). Gamma-rays irradiation: an effective method for improving light emission stability of porous silicon. Appl Phys Lett.

[11] Astrova EV, Emtsev VV, Lebedev AA (1995). Degradation of the photoluminescence of the porous silicon under the influence of gamma-radiation ^60^Co. Fizika i Tekhnika Poluprovodnikov.

[12] Astrova EV, Bитмaн PФ, Emtsev VV (1996). Influence γ–radiation on properties of porous silicon. Fizika i Tekhnika Poluprovodnikov.

[13] Lisovskyy IP, Indutniĭ IZ, Muravskaya MV (2008). Enhancement of photoluminescence of structures with nanocrystalline silicon stimulated by low-dose irradiation with γ-ray photons. Semiconductors.

[14] Jang S, Joo B, Kim S (2015). Effects of proton irradiation on Si-nanocrystal/SiO_2_ multilayers: study of photoluminescence and first-principles calculations. J Mater Chem C.

[15] Borkovskaya OY, Dmytruk ML, Konakova RV (1985). Effects of radiation-induced ordering in the layer structures on the base of A^III^ B^V^.

[16] Szekeres A, Nikolova T, Paneva A (2005). Silicon nanoparticles in thermally annealed thin silicon monoxide films. Mater Sci Enginuring B.

[17] Fok MV (1969). On the deconvolution of the complete spectra into individual contributions. J Appl Spectroscopy.

[18] Schmidt T, Lischka K, Zulehner W (1992). Excitation-power dependence of the near-band-edge photoluminescence of semiconductors. Phys Rev B.

[19] Canham LT (1990). Silicon quantum wire array fabrication by electrochemical and chemical dissolution of wafers. Appl Phys Lett.

[20] Kanzawa Y, Kageyama T, Takeoka S (1997). Size-dependent near-infrared photoluminescence spectra of Si nanocrystals embedded in SiO_2_ matrices. Solid State Commun.

[21] Dhara S, Lu C-Y, Nair K (2008). Mechanism of bright red emision in Si nanoclusters. Nanotechnology.

[22] Sato K, Hirakuri K (2006). Influence of paramagnetic defects on multicolored luminescence from nanocrystalline silicon. J Appl Phys.

[23] Takeguchi M, Furuya K, Yoshihara K (1999). Structure study of Si nanocrystals formed by electron-induced reduction of SiO_2_ at high temperature. Jpn J Appl Phys.

[24] Xi-Wen D, Takeguchi M, Tanaka M (2003). Formation of crystalline Si nanodots in SiO_2_ films by electron irradiation. Appl Phys Lett.

[25] Lisovskii IP, Litovchenko VG, Lozinskii VB, Steblovskii GI (1992). IR spectroscopic investigation of SiO_2_ film structure. Thin Solid Films.

[26] Sato K, Hirakuri K (2005). Improved luminescence intensity and stability of nanocrystalline silicon due to the passivation of nonluminescent states. J Appl Phys.

[27] Biteen JS, Lewis NS, Atwater HA (2004). Size-dependent oxygen-related electronic states in silicon nanocrystals. Appl Phys Lett.

[28] Wolkin MV, Jorne J, Fauchet PM (1999). Electronic states and luminescence in porous silicon quantum dots: the role of oxygen. Phys Rev Lett.

[29] Grunthaner FJ, Grunthaner PJ (1986). Chemical and electronic structure of the SiO_2_/Si interface. Mat Sci Rep.

